# Supplementary material for: Adverse outcomes in patients hospitalized with pneumonia at age 60 or more: A prospective multi-centric hospital-based study in India
Source: PLoS One. 2024 May 2;19(5):e0297452. doi: 10.1371/journal.pone.0297452 (PMC11065220; doi:10.1371/journal.pone.0297452)
Supplement: Attachment — Submitted filename: Authors Responses to Reviewer Comments.docx [file pone.0297452.s001.docx]

**Authors Responses to Reviewer Comments:**

We express our acknowledgement to the reviewers for their precious assessment of our work. Subsequently, we address their concerns in a point-by-point manner.

**Reviewer 1**

**Comment:**

Community acquired pneumonia (CAP) is a significant cause of morbidity among older adults. However, studies on the etiology & risk factors associated with adverse clinical outcomes mostly emerge from high income countries. This study attempts to identify the causes of CAP and to link them to clinical outcomes in a middle-income country- India, with the ultimate goal of informing policy around managing the CAP associated burden on the medical system. Overall, the study is well-designed, analysis is rigorous and conclusions are proportional to the results. Studies of this kind tend to be descriptive in nature. Nevertheless, I appreciate the authors’ attempts to provide some policy recommendations based on their data.

**Response**

We express our gratitude for your constructive and motivating feedback.

**Reviewer 2**

**Comment 1:**

The title of the manuscript is confusing because it does not clearly express the idea/conclusion/goal of the article. I strongly suggest editing/paraphrasing the first part of the title (before the colon mark). A prospective title could be: “Adverse outcomes in patients hospitalized with pneumonia at age 60 or more: a prospective multi-centric hospital-based study in India.

**Response:** We thank the reviewer for bringing this to our attention. The mentioned section has been appropriately revised in accordance with the reviewer's feedback. Therefore, the title updated in the revised manuscript as “*Adverse outcomes in patients hospitalized with pneumonia at age 60 or more: a prospective multi-centric hospital-based study in India*”.

**Comment 2: Section-** Introduction

Introduction line 72 needs the reference that it is talking about.

**Response:** We express gratitude to the reviewer for bringing this to our attention, and we have now incorporated the relevant reference in the revised manuscript at line 73, that is *Ref no. 6***:** *Lafond KE, Porter RM, Whaley MJ, Suizan Z, Ran Z, Aleem MA, et al. Global burden of influenza-associated lower respiratory tract infections and hospitalizations among adults: A systematic review and meta-analysis. PLoS Med. 2021;18: 1–17. doi:10.1371/JOURNAL.PMED.1003550*

**Comment 3: Section-** Introduction

Line 76-77: “CAP hospitalization and mortality in middle income countries, however, can be dramatically higher”- needs a reference, or a reason if it is a mere speculation.

**Response:** We have addressed the reviewer's concern by including the relevant references into lines 77-78 of the revised manuscript. The relevant ref nos. are Ref no. 9 and Ref no.10.

Ref no. 9: *Austin S, Murthy S, Wunsch H, Adhikari NKJ, Karir V, Rowan K, et al. Access to urban acute care services in high- vs. middle-income countries: An analysis of seven cities. Intensive Care Med. 2014;40: 342–352. doi:10.1007/S00134-013-3174-7/TABLES/5.*

Ref no. 10: *Salluh JIF, Kawano-Dourado L. Implementing the severe community-acquired pneumonia guidelines in low- and middle-income countries. Intensive Care Med. 2023;49: 1392–1396. doi:10.1007/S00134-023-07220-7/METRICS.*

**Comment 4: Section-** Introduction and method

Line 151: CRUB-65 scoring is mentioned twice. Please correct it. Also, please define “CRUB-65 scoring” briefly in the method section.

**Response:** We have addressed the reviewer's concern and corrected it accordingly in line 152 of the revised manuscript. Furthermore, we have addressed the reviewer's concern on CURB-65 scoring and briefly defined the "CRUB-65 scoring" in the method section in lines 152-155.

**Comment 4:**

The writing format of ‘P value’ is inconsistent. Also, I suggest not to use a dash (-) between ‘p value’ and the number. For example: line 184, 201, 205, etc. Please revise to make it consistent.

**Response:** Thank you for your valuable feedback. We accept the inconsistency in the writing format of 'P value'. We revised the manuscript to ensure a consistent format for 'p value' without the use of a dash as per the reviewer’s recommendation.

**Comment 5:**

There are only figures in the document, whereas authors can make easily make more figures to represent some important data. For example: the type of virus detected in four different sites can be presented as individual “pie charts”, which is a key outstanding information included in table 1. Similarly, the data shown in Table 2 can also be presented as pie chart for each type of facilities. Figures are always helpful for readers to get the message and compare among different sites**.**

**Response:**

We appreciate the time and effort you dedicated to reviewing our manuscript. Your suggestion to represent the type of virus detected in four different sites as individual "pie charts" and to present the data in Table 2 as pie charts for each type of facility is really thoughtful and may conveying information effectively to readers.

However, we would like to clarify that we try to provide a comprehensive overview and we intentionally opted to present all relevant data in a single consolidated table rather than dispersing it multiple figures. Our aim was to represent the interrelationships among different variables.
